# Hyperemesis gravidarum in the primary care setting: cross-sectional study of GPs

**DOI:** 10.3399/BJGPO.2021.0119

**Published:** 2021-12-08

**Authors:** Melanie Nana, Holly Morgan, Haroon Ahmed, Catherine Williamson

**Affiliations:** 1 Department of Obstetric Medicine, St Thomas’ Hospital, London, UK; 2 Department of Cardiology, King’s College London, London, UK; 3 Division of Population Medicine, Cardiff University School of Medicine, Cardiff, UK; 4 Department of Life Course Sciences, King’s College London, London, UK

**Keywords:** hyperemesis gravidarum, education, pregnancy, antiemetics, primary health care, general practice

## Abstract

**Background:**

Hyperemesis gravidarum (HG), if untreated, can lead to malnutrition, dehydration, and Wernicke’s encephalopathy. Foetal complications include low birth weight and neurodevelopmental delay. Recent evidence supports increased rates of termination of pregnancy and suicidal ideation. Drivers included difficulty in accessing medications, which thus contributed to poor perception of care.

**Aim:**

To identify factors that may influence prescribers’ confidence and knowledge regarding pharmacological therapy for HG.

**Design & setting:**

Cross-sectional study of qualified GPs and GP trainees in Wales.

**Method:**

Distribution of a 22-item online survey. Statistical analysis was carried out using SPSS.

**Results:**

In total, 241 responses were received, with 216 included in the analysis (59% qualified GPs, 41% GP trainees). In total, 93% of responders correctly identified cyclizine as being safe in pregnancy, but no other drug recommended in the Royal College of Obstetrics and Gynaecology guidance was considered safe by more than 58%. Those reporting higher confidence levels in managing HG were more likely to correctly report guideline-recommended drugs as safe in pregnancy (*P* = 0.04). Additional qualifications related to obstetrics and gynaecology (O&G) and/or prior clinical experience increased confidence levels (*P* = 0.0001 and *P* = 0.0002, respectively). Only 19% of participants routinely screened for signs of mental health complications, and prior experience or education did not increase likelihood of this happening. The majority of participants (87%) would like additional education and/or access to evidence-based resources.

**Conclusion:**

This study demonstrates a demand for improved dissemination of evidence-based education on HG to support those working in primary care. The extent to which HG is covered in pre-existing educational programmes should also be revisited.

## How this fits in

To the authors' knowledge this is the first study to explore the factors that influence the management of those caring for women with HG in primary care. The study demonstrates the need to better support clinicians by providing access to education and comprehensive guidance. The extent to which HG is covered in pre-existing educational programmes should also be revisited. Work is now ongoing to develop e-learning material and to update and publicise guidance.

## Introduction

HG describes nausea and vomiting in pregnancy excessive enough to result in dehydration and weight loss.^1^ It complicates between 0.3%–3.6% of pregnancies, which equates to between 1900–23 000 affected women in the UK per year.^1–3^

Presentation includes severe intractable vomiting, often associated with >5% weight loss, dehydration, and electrolyte imbalance, with symptoms typically starting between the seventh and ninth week of pregnancy.^3,4^ Untreated, it may result in complications secondary to malnutrition and dehydration. Maternal complications include electrolyte disturbance (15%–28%), Wernicke’s encephalopathy (secondary to thiamine deficiency; <1%), and susceptibility to thrombus.^5,6^ Mental health complications include increased rates of anxiety (46%), depression (48%), and suicidal ideation (7%).^7–13^ Effects on the foetus include a four-fold increased risk of low birthweight and preterm birth, and three-fold increased odds of neurodevelopmental delay.^14,15^

Initial management is typically carried out in primary care comprising use of first line anti-emetics (antihistamines and phenothiazines).^16^ Practitioners should assess a woman’s mental health status and refer for psychosocial support if necessary.^16^ Timely community-based treatment, including prompt pharmacological therapy where required, should be offered to avoid complications.^17,18^ Inpatient management should be considered in women who, despite treatment with oral anti-emetics, have persistent vomiting, clinical evidence of dehydration, weight loss of >5% of their body weight, or a confirmed or suspected comorbidity.^16^

In a recent survey of >5000 women with HG, 40% perceived their experience in primary care to be poor or extremely poor in terms of HG management.^13^ These women were more likely to terminate a wanted pregnancy as a consequence of HG or experience suicidal ideation. They were more likely to have struggled accessing medication, with 48% of those taking medications having to actively request it as opposed to being offered it. Qualitative analysis confirmed difficulty accessing appropriate treatment with a negative impact on the ability of affected women to look after family and earn a living. The study also revealed marked variations in the attitude of healthcare professionals towards women with HG.

A number of factors may contribute to a delay in timely prescription of anti-emetics in pregnancy. The 1960s thalidomide disaster rendered all medications used in pregnancy suspect of teratogenicity.^19^ As such, practitioners exercise significant caution prescribing in pregnancy. However, a Cochrane review and other systematic reviews and meta-analyses have now reported on the safety and efficacy of many anti-emetics in pregnancy with no increased risk of teratogenesis or other adverse outcomes, and so a risk–benefit decision should be made between prescribing such medications and the risks of untreated HG.^20,21^

GPs see a large number of patients every day, making decisions on a wide range of medical problems. While it is unreasonable to expect detailed sub-specialty knowledge, it is imporant that GPs are provided with evidence-based information regarding management of such patients, and that opportunities are available to those wishing to develop knowledge in this area so that patients can be supported in gaining timely access to treatment. Available guidance includes The Royal College of Obstetricians and Gynaecologists (RCOG) Green-top guideline and the National Institute for Health and Care Excellence Clinical Knowledge Summary.^16,22^

This study aimed to explore the confidence of GPs in managing HG patients in Wales and their knowledge surrounding pharmacological therapy. It aimed to identify factors that may influence these and explore methods by which GPs could be supported in terms of education, resources, and continued professional development.

## Method

This cross-sectional study utilised a 22-item online survey developed using the online platform surveymonkey.com. The survey was piloted by the Pregnancy Sickness Support charity chairman and two GPs based in Wales (one trainee and one qualified GP, both with an interest in medical education) with feedback incorporated. Included questions were either multiple choice or open comment (Supplementary Table S1).

An invitation to complete the survey was sent out to qualified GPs and GP trainees, between 19 January and 5 March 2020, via a number of avenues. Emails were sent to all postgraduate centres and the head of the School of General Practice at Health Education and Improvement Wales to enable dissemination to GP trainees. The link was also sent to regional primary care representatives for dissemination to qualified GPs, and was posted on social media.

The data requested included demographic information; prior experience in O&G; previous education relating to HG; access to and attendance at continued professional development education opportunities; confidence managing patients with HG; practitioner knowledge regarding pharmacotherapy; services available to support the management of such patients; and data regarding whether further education opportunities would be valuable and in what form. All data were collected anonymously and voluntarily. All qualified GPs and GP trainees working in Wales were eligible to participate. Participants required access to the internet. Other allied healthcare professional and students were excluded.

It was not compulsory for participants to answer every question, thus the total response number for each question varies. Mean confidence scores were calculated by awarding 1–4 points depending on answer (1 = not at all confident, 2 = not so confident, 3 = somewhat confident, 4 = very confident). Data are presented as percentages and raw numbers. For confidence scores, means have been used rather than medians to highlight differences between groups. Statistical analysis was carried out using SPSS (software version 11.0, IBM); Mann–Whitney U and 2 tests were used for non-parametric data, and ANOVA for multiple groups. A value of *P*<0.05 was considered statistically significant.

## Results

### Demographics

**Figure 1.**
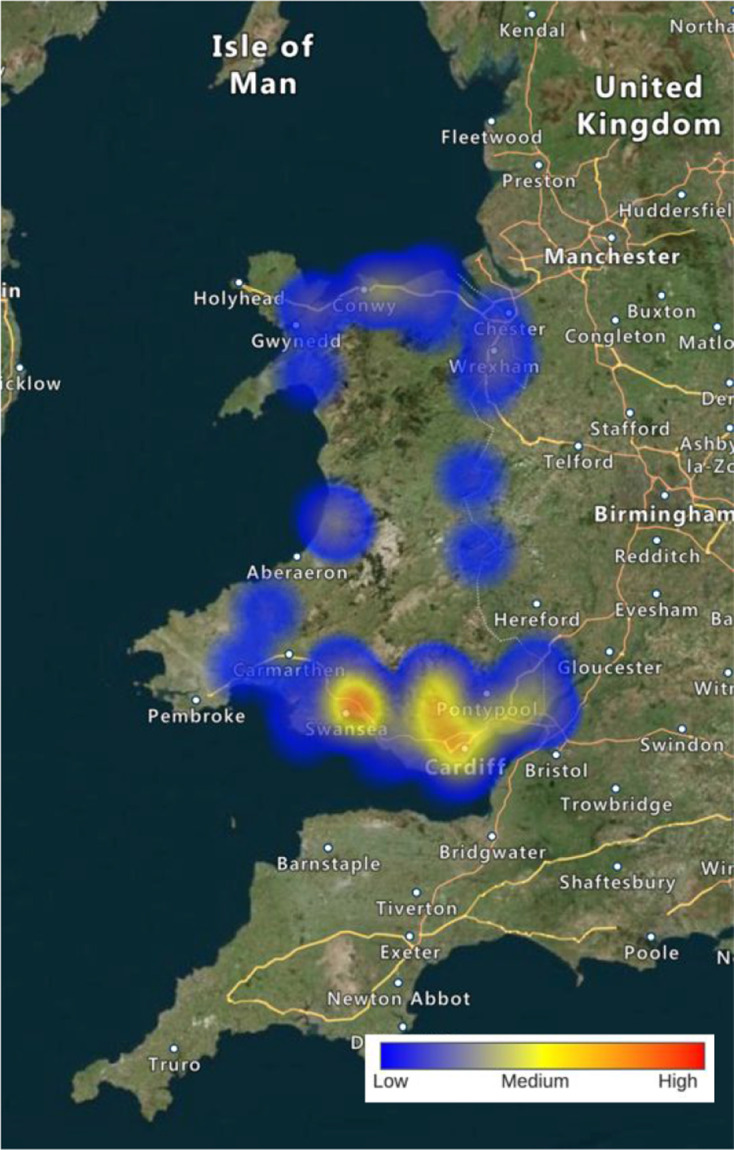
Heat map demonstrating geographical distribution of participants

A total of 241 responses were received. Of these, 13 were largely incomplete and 12 were filled in by other allied healthcare professionals. It was felt that, because the overall number of responses from allied healthcare professionals was small and there was not representation from more than three members of one allied healthcare group, these responses should be removed. Of the remaining 216 responses, 59% (*n* = 128/216) were completed by fully qualified GPs and 41% (*n* = 88/216) by GP trainees. In terms of geographical location, 26% (*n* = 57/216) of participants reported being from rural practices, 39% (*n* = 85/216) suburban, and 34% (*n* = 74/216) from urban practices (percentages do not total 100 due to rounding; Figure 1 and Supplementary Table S2).

### Experience and education

**Figure 2.**
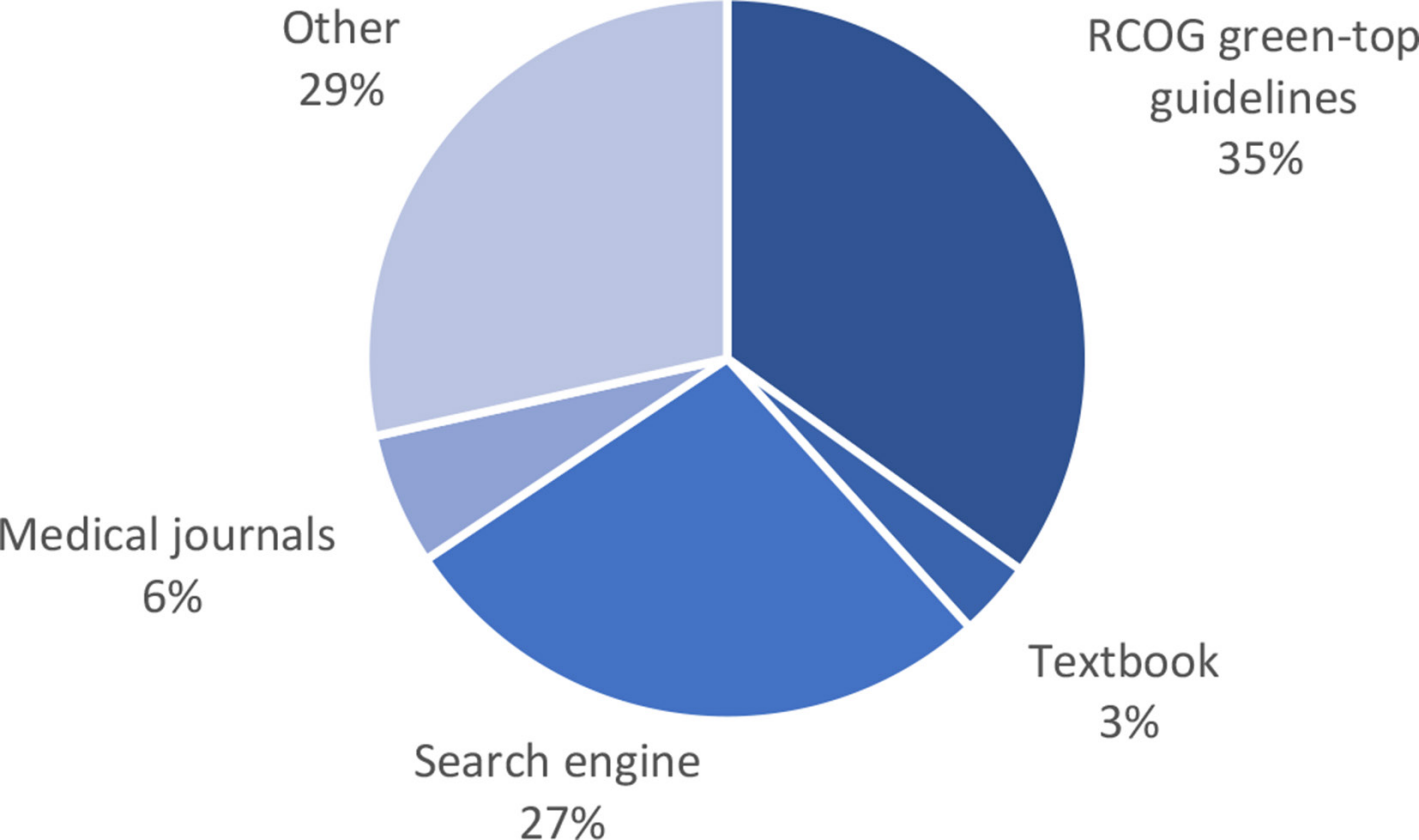
Breakdown of where participant would access further information on HG. ‘Other’ included the British Medical Journal resources, the British National Formulary, GP notebook, National Institute for Health and Care Excellence resources, patient information websites, and the website of a widely used GP educational resource (the ‘Red Whale’ website).

In total 69% (*n* = 150/216) of responders reported having at least one additional postgraduate qualification or prior clinical experience related to O&G. The nature of the qualification or experience is illustrated in Supplementary Figure S1. 38% (*n* = 82/213) of participants reported having received previous teaching focused on HG. Open box comments revealed that this had taken place primarily in medical school or as part of departmental teaching on O&G placements (Supplementary Table S2). Participants reported using a variety of resources to access further information regarding HG (Figure 2).

Regarding access to continued professional development (CPD), 92% (*n* = 198/216) of participants reported that they attended or had access to community or GP teaching. 23% (*n* = 50/213) routinely attend regional or national conferences, including the ‘Hot Topics GP update course’ and Royal College of General Practitioners conferences (Supplementary Table S2).

Qualified GPs were more likely to attend regional or national conferences than GP trainees (*P* = 0.01), and geography did not affect this outcome. There was no association between grade of training or geography and any other education outcome (Supplementary Table S2).

### Confidence

**Figure 3.**
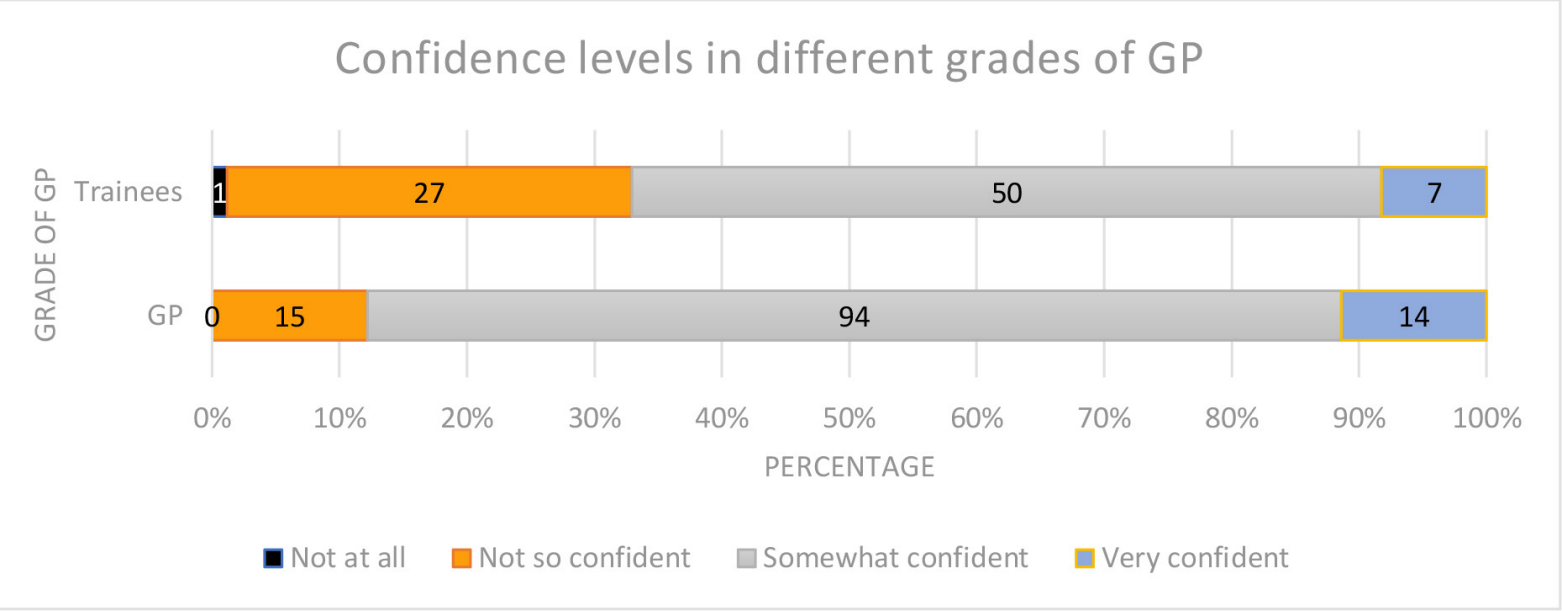
Participants confidence levels with regards to managing women with HG

Participants were asked how confident they felt managing patients with HG (Figure 3).

The mean confidence scores for qualified GPs (3.0/4) were statistically significantly higher than the scores of GP trainees (2.7/4), (*P* = 0.002). Those who had an additional qualification or prior clinical experience related to O&G and those who had received previous teaching on HG were also significantly more confident in managing HG, (*P* = 0.0001 and *P* = 0.0002, respectively) (Table 1).

**Table 1.** Relationship between additional qualifications or training and confidence levels and likelihood of screening for mental health consequences in HG.

	Confidence in managing HG (score/4)	Routinely screen for mental health problems
Additional qualifications or prior clinical experience related to O&G		
Yes	3.0	19% (27/145)
No	2.6	19% (12/64)
*P* value	0.0001^a^	1.0
Attendance or access to community/ GP teaching		
Yes	2.9	18% (35/192)
No	3.1	24% (4/17)
*P* value	0.21	0.59
Routine attendance at regional or national conferences		
Yes	3.0	22% (11/49)
No	2.9	18% (28/160)
*P* value	0.35	0.44
Previous teaching on HG		
Yes	3.1	17% (13/78)
No	2.8	20% (26/131)
*P* value	0.0002^a^	0.31

^a^Statistically significant.

### Management of HG including prescription of pharmacotherapy

Participants were asked what signs and symptoms would determine their decision to admit a patient with HG. The survey question was formatted such that they could ‘tick all that applied’. In total, 214 participants responded to the question; the most common reasons for admission included clinical evidence of dehydration, inability to tolerate oral anti-emetics, and ketonuria (Supplementary Figure S2).

### Safety and prescription of medications

Participants were asked to select answers regarding the safety and prescription of the commonly used medications in HG. Of the first line drugs recommended by the RCOG Green-top guidelines, cyclizine was the only drug that a large proportion of responders (93%) were comfortable prescribing in pregnancy. No other drug recommended in the guideline was considered safe by more than 58% of responders (Supplementary Table S3). For the remaining three first line recommended anti-emetics (prochlorperazine, promethazine, and chlorpromazine), 15–57% of participants felt comfortable prescribing them. In addition, 5–46% of responders believed that the first line recommended anti-emetics should only be prescribed in secondary care. In total, 59% of participants reported ondansetron as being safe in pregnancy, with 52.2% feeling comfortable prescribing it in primary care. (Supplementary Table S3).

In total, 47% of participants believed that thiamine, recommended to prevent Wernicke’s encephalopathy, is safe throughout pregnancy; 19% believed it should not be prescribed in pregnancy; and 39% believed it should be reserved for secondary care prescription only (Supplementary Table S3).

Responders with higher confidence levels in managing HG were significantly more likely to report guideline-recommended drugs being safe in pregnancy (*P* = 0.04) and significantly less likely to report that guideline-recommended medications should not be prescribed in pregnancy (*P* = 0.04). There were trends towards increased numbers of participants reporting guideline-recommended medications being safe in pregnancy in those with additional qualifications or clinical experience, and in those who had previous teaching on HG (Table 2).

**Table 2.** Participant knowledge and comfort in prescribing first line anti-emetics and all drugs recommended in guidelines

	Safety of medications in pregnancy	Comfort of physician in prescribing in primary care
Participants reporting first line anti-emetics being safe throughoutpregnancy, %	Participants reporting all guideline-recommended drugs being safe inpregnancy, %	Participants reporting first line anti-emetics not being safe in pregnancy, %	Participants reporting that guideline-recommended medications should not be prescribed in pregnancy, %
Confidence levels				
Not at all/not so confident	27%	28%	7%	30%
Somewhat confident	52%	50%	5%	17%
Very confident	55%	57%	5%	17%
*P* value	0.09	0.04^a^	0.49	0.04^a^
Additional qualifications/additional clinical experience				
Yes	52%	50%	5%	19%
No	37%	37%	7%	21%
*P* value	0.24	0.11	0.23	0.38
Previous teaching on hyperemesis gravidarum (HG)				
HG teaching	52%	49%	5%	16%
No HG teaching	43%	43%	6%	21%
*P* value	0.34	0.29	0.27	0.18

^a^Statistically significant. HG = hyperemesis gravidarum.

Overall, 19% (*n* = 39/207) of participants stated that they routinely screen women with HG for signs of mental health problems. The likelihood of performing screening was not affected by having obtained additional education (Table 1). Only 7% (*n* = 12/164) of participants had access to mental health counselling (four working in urban practices, five in suburban, and three in rural practices).

### Additional education

In total, 87% (*n* = 141/163) of participants reported that they would like to receive additional education or have access to additional resources. In terms of the educational material that they would like to receive (participants were able to ‘tick all that apply’), the most common responses included online e-learning (117 participants), online guidelines (108 participants), and teaching integrated into the GP training programme (87 participants).

## Discussion

### Summary

This study collated responses from 216 GPs of varying grade and geographical distribution across Wales, consistent with response numbers from similar studies.^23,24^ In total, 69% of participants had at least one postgraduate qualification or prior clinical experience relating to O&G. Despite this, only 38% reported receiving previous teaching focused on HG. Grade of training and geography did not affect likelihood of having additional experience, teaching, or access to CPD.

The majority of participants (93%) felt comfortable prescribing cyclizine in pregnancy. The proportion of responders that felt comfortable prescribing the other three first line recommended anti-emetics (prochlorperazine, promethazine, and chlorpromazine) was 57%, 46%, and 15%, respectively. Clinical evidence of dehydration, inability to tolerate oral anti-emetics, and ketonuria represented the most common reasons for a participant to decide to admit a patient.

Those who reported increased levels of confidence in managing HG were significantly more likely to report all guideline-recommended drugs being safe in pregnancy and significantly less likely to report that guideline-recommended medications should not be prescribed in pregnancy (Table 2). Prior additional qualifications or clinical experience related to O&G and previous teaching on HG were identified as factors that increased confidence levels. These factors additionally supported trends towards participants being more likely to report guideline-recommended medications being safe in pregnancy (Table 2).

Only 19% routinely screened women with HG for signs of mental health problems, with no increased likelihood of screening occurring in those with additional qualifications, prior clinical experience, or previous teaching on HG (Table 1). Only 7% of participants reported having access to mental health counselling.

There was a clear demand for additional education and access to additional resources. The majority of participants expressed a preference for learning materials in the form of online e-learning, online guidelines, and teaching integrated into the GP training programme.

### Strengths and limitations

To the authors' knowledge, this is the first study to explore the factors that influence the management of those caring for women with HG in primary care. Online distribution of the survey, via a number of avenues, facilitated the acquisition of responses from a number of GPs, with representation from a range of training grades and geography. As with all survey-based studies, this study is limited by the risk of responder bias and reliance on participants fully understanding each question, and thus providing ‘conscientious responses’. Responders were not obliged to complete each question; a degree of ‘survey fatigue’ was observed, with a degree of participant drop out throughout the survey. The survey nature of the work does not allow the authors to further explore reasons for increased completion of certain questions over others. This study focused on GPs, but the authors recognise the importance of input from the multidisciplinary team in the management of women with HG. As such, it will be important for future work to also focus on the knowledge and confidence in managing HG of the wider multidisciplinary team.

### Comparison with existing literature

Extensive literature exists describing the burden of HG on patient’s physical and mental health.^5,9,10^ In addition, HG is the most common reason for hospitalisation during pregnancy, the subsequent financial burden on the health service being estimated at approximately 36.5 million pounds per year.^25–28^

A large population-based cohort study identified 37 856 women who experienced nausea and vomiting in pregnancy (NVP) or HG. Of these, 6390 had a hospital admission prior to 20 weeks and 2425 after 20 weeks’ gestation.^29^ Only 38% and 23%, respectively, had evidence of primary care prescription of anti-emetics before admission. Challenges in accessing pharmacotherapy for HG in primary care are well documented, with 48% of women taking medications in a recent study having had to actively request it, despite it being acknowledged that early treatment may prevent admission.^11,13,30,31^ This study's findings propose a possible rationale for those findings, as it has demonstrated lack of confidence in prescribing first line anti-emetics in pregnancy. National guidelines for the managements of NVP and HG were published in 2016, providing recommendations on the management of such women; despite this, only 39% of participants in this study felt comfortable prescribing first line recommended treatments, and only 35% of participants refer to these guidelines when seeking further information regarding HG.^16^

This study reported that 59% of participants consider ondansetron as being safe in pregnancy, with a 52% feeling comfortable prescribing it in primary care. A controversial statement issued by the European Medicines Agency in August 2019 recommended that ondansetron should not be prescribed in the first trimester of pregnancy due to concerns regarding the risk of orofacial malformations.^32^ The UK teratology information service have since issued an official response statement summarising the increased risk of orofacial clefts equates to an additional three cases per 10 000 pregnancies exposed to ondansetron.^33^ As such, ondansetron should be reserved as a second line agent but not exclusively avoided in the first trimester of pregnancy; this statement is supported by the RCOG guidelines.^16^ In many cases, the benefit of ondansetron will outweigh the risks, and it is therefore reassuring that over half of GPs in this study were comfortable prescribing it in primary care.

Only 19% of participants were screening for mental health problems. Previously published literature supports a lack of attention being paid towards the detrimental mental health consequences of HG. Previous studies exploring patient experience describe feelings of not being believed and stigma being displayed towards those with HG.^9,11,34^ In this study, additional qualifications or prior clinical experience related to O&G, nor previous teaching on HG, increased the likelihood of a participant screening for mental health problems, suggesting that current education surrounding HG does not adequately cover the psychiatric burden of the condition.

In total, 185 participants selected ketonuria as an indication for deciding to admit a patient with HG to hospital. While the presence of ketones reflects starvation and is therefore a possible surrogate marker of severity in HG, it is increasingly being recognised that its use as a ‘gatekeeper’ for admission is flawed since this is not the only manifestation of severity of HG and does not reflect degree of dehydration.^35^ As such, use of ketone measurement will be excluded in the next iteration of the RCOG guidance.

### Implications for practice

This study demonstrates the need to support those in primary care in looking after women with HG by providing access to education and comprehensive guidance. The small proportion of GPs having confidence in prescribing guideline-recommended treatments, as well as low numbers reporting referring to guidance when requiring further information on HG, suggests the guidance needs to be better publicised and more accessible. Recommended resources are outlined in Table 3.

**Table 3.** List of recommended resources for healthcare professionals.

Recommended resources
RCOG	The Management of Nausea and Vomiting of Pregnancy and Hyperemesis Gravidarum - Green-top Guideline
National Institute for Health and Care Excellence	Nausea/vomiting in pregnancy- Clinical Knowledge Summary
UK Teratology Information Service	Treatment of Nausea and Vomiting in Pregnancy
UpToDate	Nausea and vomiting of pregnancy: Treatment and outcomes
Red Whale	https://www.gp-update.co.uk/Latest-Updates/Nausea-and-vomiting-in-pregnancy
Pregnancy Sickness Support charity	Section for Healthcare Professionals
HER Foundation	Section for Healthcare Providers

Confidence, and thus appropriate selection of safe medications, was increased in those with previous postgraduate qualifications or clinical experience related to O&G, and previous teaching on HG. Participants unanimously reported a hunger for additional education, suggesting online e-learning as preferred method of delivery which should prompt further work in developing such resources.

Only a proportion undertaking additional qualifications or with clinical experience related to O&G had undertaken previous teaching on HG. In addition, previous education made no difference on likelihood of screening for mental health problems, suggesting that syllabus’ and curriculums should be revisited to ensure that they adequately cover this profound disease.
